# Phenotypic Heterogeneity in a DFNA20/26 family segregating a novel *ACTG1* mutation

**DOI:** 10.1186/s12863-016-0333-1

**Published:** 2016-02-01

**Authors:** Yongyi Yuan, Xue Gao, Bangqing Huang, Jingqiao Lu, Guojian Wang, Xi Lin, Yan Qu, Pu Dai

**Affiliations:** Department of Otolaryngology, Chinese PLA General Hospital, Beijing, 100853 People’s Republic of China; Department of Otolaryngology, Hainan Branch of PLA General Hospital, Sanya, 572000 People’s Republic of China; Department of Otolaryngology, Emory University School of Medicine, Atlanta, GA 30322-3030 USA; Third hospital of Hebei Medical University, Shijiazhuang, Hebei Province 050051 People’s Republic of China

**Keywords:** Autosomal dominant nonsyndromic hearing impairment, Mutation, *ACTG1*, *GJB2*, Digenic inheritance

## Abstract

**Background:**

Genetic factors play an important role in hearing loss, contributing to approximately 60 % of cases of congenital hearing loss. Autosomal dominant deafness accounts for approximately 20 % of cases of hereditary hearing loss. Diseases with autosomal dominant inheritance often show pleiotropy, different degrees of penetrance, and variable expressivity.

**Methods:**

A three-generation Chinese family with autosomal dominant nonsyndromic hearing impairment (ADNSHI) was enrolled in this study. Audiometric data and blood samples were collected from the family. In total, 129 known human deafness genes were sequenced using next-generation sequencing (NGS) to identify the responsible gene mutation in the family. Whole Exome Sequencing (WES) was performed to exclude any other variant that cosegregated with the phenotype.

**Results:**

The age of onset of the affected family members was the second decade of life. The condition began with high-frequency hearing impairment in all family members excluding III:2. The novel *ACTG1* c.638A > G (p.K213R) mutation was found in all affected family members and was not found in the unaffected family members. A heterozygous c.638A > G mutation in *ACTG1* and homozygous c.109G > A (p.V37I) mutation in *GJB2* were found in III:2, who was born with hearing loss. The WES result concurred with that of targeted sequencing of known deafness genes.

**Conclusions:**

The novel mutation p.K213R in *ACTG1* was found to be co-segregated with hearing loss and the genetic cause of ADNSHI in this family. A homozygous mutation associated with recessive inheritance only rarely co-acts with a dominant mutation to result in hearing loss in a dominant family. In such cases, the mutations in the two genes, as in *ACTG1* and *GJB2* in the present study, may result in a more severe phenotype. Targeted sequencing of known deafness genes is one of the best choices to identify the genetic cause in hereditary hearing loss families.

**Electronic supplementary material:**

The online version of this article (doi:10.1186/s12863-016-0333-1) contains supplementary material, which is available to authorized users.

## Background

Nonsyndromic hearing impairment (NSHI) is a common sensory defect in humans, and most cases of NSHI are attributable to genetic factors [1]. The inheritance patterns of NSHI include autosomal dominant, autosomal recessive, X-linked, and mitochondrial inheritances. Autosomal dominant deafness accounts for approximately 20 % of cases of hereditary hearing loss [2]. To date, more than 30 genes and 50 genetic loci have been implicated in autosomal dominant NSHI (ADNSHI) (http://hereditaryhearingloss.org/). These deafness genes encode a multiplicity of proteins that function in various cell types, structures, and processes in the cochlea [3].

Diseases with autosomal dominant inheritance exhibit pleiotropy, different degrees of penetrance, and variable expressivity. ADNSHI is difficult to distinguish phenotypically [2]. In contrast to autosomal recessive nonsyndromic hearing loss, most patients with ADNSHI show large variation in age of onset; hearing impairment often begins before the age of 20 years and progresses gradually. However, deafness, autosomal dominant nonsyndromic sensorineural 4 (DFNA4,OMIM:600652), DFNA9 (OMIM:601369), and DFNA10 (OMIM:601316) are associated with hearing impairment starting in the third and fourth decades of life [2]. Additionally, the ADNSHI phenotypes vary and are divided into low-frequency, mid-frequency, high-frequency, and all-frequency hearing impairment [4]. Thus, in clinical molecular diagnosis, the hearing loss phenotype in a patient can help to select a limited number of genes for mutational analysis.

Digenic mutations leading to hearing loss have been reported in previous studies and include *GJB2/GJB6* [5], *GJB2/GJB3* [6], and *KCNJ10/SLC26A4* [7]. However, all exhibit recessive/recessive inheritance, while dominant/dominant and dominant/recessive inheritances are rare.

We herein report a family with eight individuals affected by sensorineural hearing loss. We used next-generation sequencing (NGS) to analyse 129 known deafness genes and identify the responsible gene mutation in the family. Whole exome sequencing (WES) was performed to exclude any other variant that cosegregated with the phenotype. The results identified one novel mutation, c.638A > G [p.K213R],in *ACTG1* in this family. A dominant mutation co-acting with a recessive mutation (heterozygous c.638A > G in *ACTG1* and homozygous c.109G > A in *GJB2*) was found in one sibling.

## Results

### Clinical presentation of the family

**Fig. 1:**
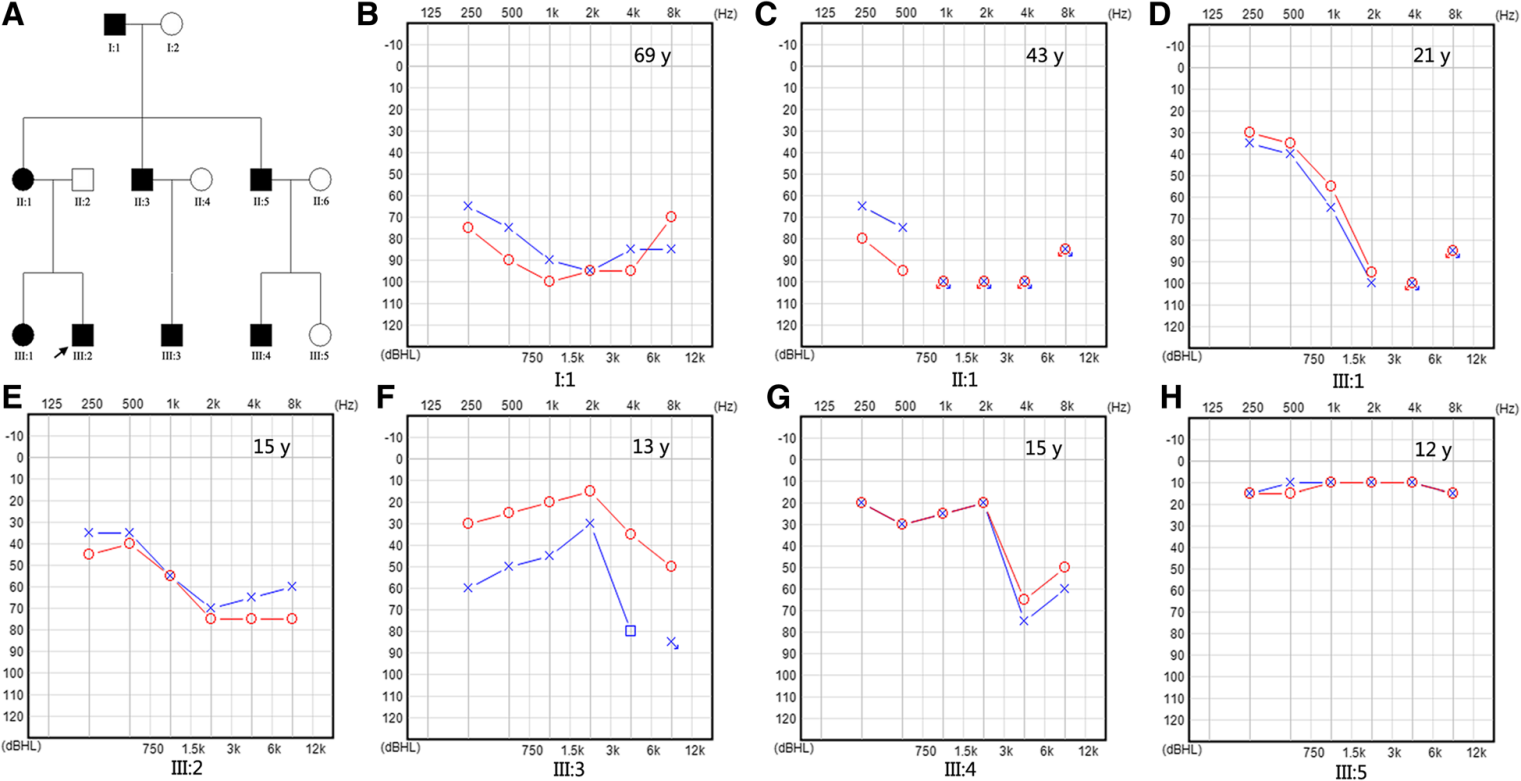
Combined figure. **a** Pedigree of the family with ADNSHL. Affected family members are denoted in black. The arrow indicates the proband. **b–h** udiogram of the family members

The family evaluated in this study included eight affected individuals and five unaffected members (Fig. 1a). Audiograms of the affected individuals revealed progressive, bilateral, sensorineural hearing loss that began at high frequencies. The degree of hearing loss increased with age, with threshold shifts ultimately seen at all frequencies. After the third decade of life, the progression of hearing loss led to profound hearing loss across all frequencies in the affected family members. The age of onset of hearing loss was during the second decade of life (Table 1). However, the proband, III:2, a 15-year-old boy, was born with hearing loss with a mean hearing threshold of approximately 60 dB (Fig. 1e).

**Table 1:** Phenotypes and genotypes of the family members

Family member	Sex	Age (years)	Genotype		Phenotype		
			*GJB2*	*ACTG1*	Age of onset (years)	PTA (Left) (dB)	PTA(Right) (dB)
1 (I:1)	M	69	N	HT c.638A > G	15	86.25	95.00
2 (I:2)	F	68	HT c.109G > A	N	/	/	/
3 (II:1)	F	43	HT c.109G > A	HT c.638A > G	17	>93.75	>98.75
4 (II:2)	M	44	HT c.109G > A	N	/	/	/
5 (II:3)	M	41	N	HT c.638A > G	14	/	/
6 (II:4)	F	40	N	N	/	/	/
7 (II:5)	M	36	N	HT c.638A > G	16	/	/
8 (II:6)	F	37	N	N	/	/	/
9 (III:1)	F	21	HT c.109G > A	HT c.638A > G	19	>76.25	>71.25
10 (III:2)	M	15	HM c.109A > G	HT c.638A > G	Birth	56.25	61.25
11 (III:3)	M	13	N	HT c.638A > G	12	51.25	23.75
12 (III:4)	M	15	N	HT c.638A > G	15	37.50	35.00
13 (III:5)	F	12	N	N	/	10.00	11.25

*M* male, *F* female, *N* normal, *HT* heterozygous, *HM* homozygous, *PTA* pure-tone audiometry

A CT scan of the temporal bone in the proband excluded inner ear malformation. Physical examination of all family members revealed no signs of systemic illness or dysmorphic features. None of the affected individuals displayed tinnitus.

### Mutation detection and analysis

**Fig. 2:**
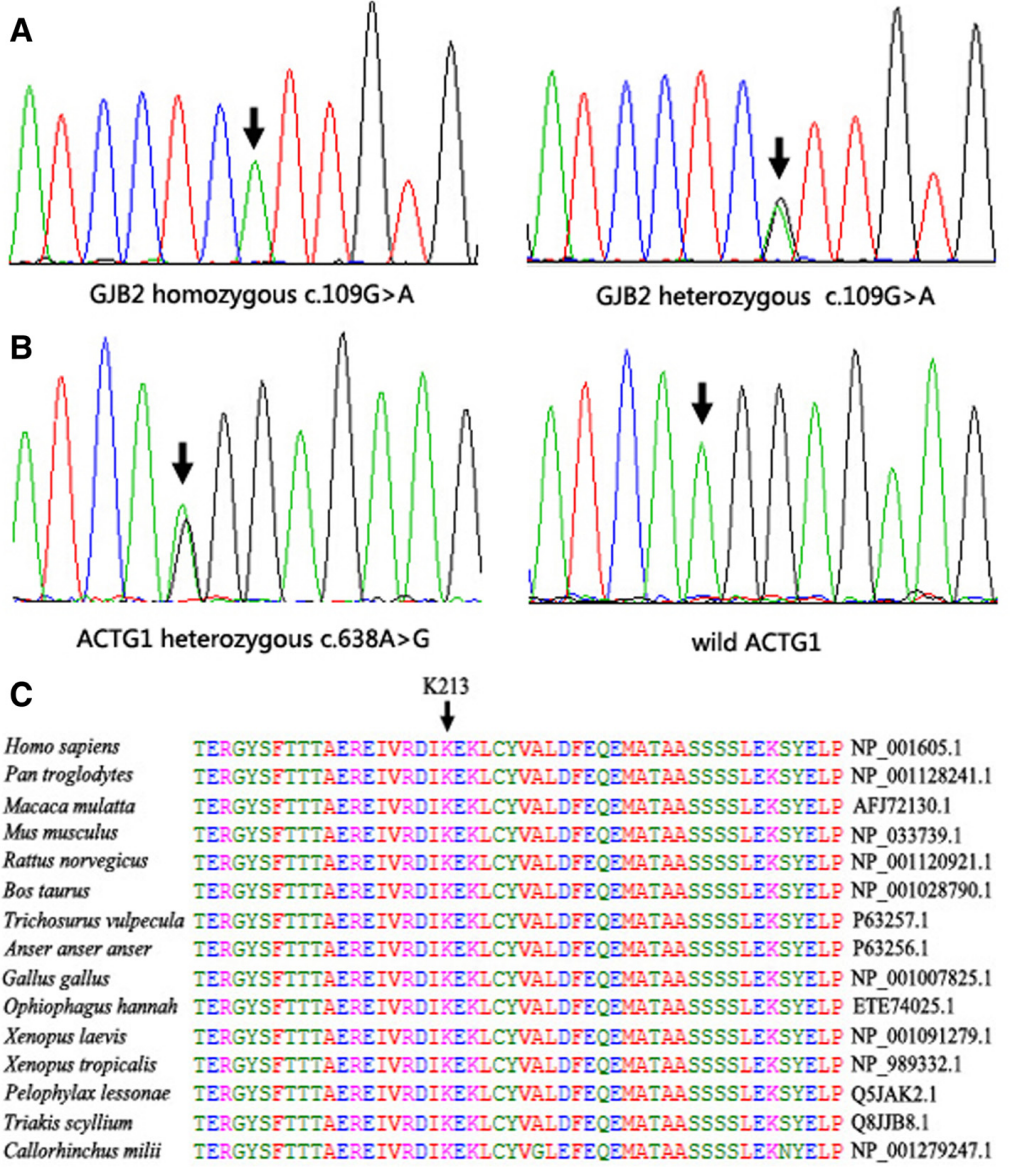
Mutation detection and conservation analysis. **a** *GJB2* mutation analysis. Sequencing results show that the homozygous c.109G > A was found in III:2 and that the parents exhibited heterozygous c.109G > A. **b** *ACTG1* mutation analysis. Sequencing results show that heterozygous c.638A > G was found in all affected family members and that wild-type *ACTG1* was found in the unaffected members. **c** Protein alignment shows conservation of the K213 residue of*ACTG1* across 15 species

First, mutations of the common deafness genes *GJB2*, *SLC26A4*, and *mtDNA* 12SrRNA were investigated in the affected family members by sequencing. For *GJB2*, the proband (III:2) was homozygous for the c.109G > A (p.V37I) mutation, while his parents were heterozygous for c.109G > A (Fig. 2a, Table 1). The c.109G > A mutation in *GJB2* did not cosegregate with the phenotype in this family. Then we performed the targeted sequencing of 129 known deafness genes in individuals I:1,I:2,II:1,II:2,II:3,II:6 and III:6.

We identified a novel mutation (c.638A > G [p.K213R]) in exon 4 of *ACTG1* in the affected family members. This mutation results in a lysine to arginine substitution at position 213 in ACTG1. Sanger sequencing revealed that all of the affected family members were heterozygous for this mutation, while the mutation was not observed in the unaffected family members (Fig. 2b, Table 1). The *ACTG1* c.638A > G mutation was not detected in the normal hearing controls.

The lysine at position 213 in ACTG1 is conserved across 15 species, as depicted in Fig. 2c. Both PolyPhen-2 and MutationTaster predicted that *ACTG1* c.638A > G (p.K213R) was a damaging mutation.

To exclude any other variant that cosegregated with the phenotype, whole exome sequencingwas performed. The proband and his parents (III:2, II:1, and II:2) were examined. For each sample, we obtained approximately 4.0–5.3 Gb of data after whole exome sequencing. The data mapped to the targeted region had a mean depth of 135.12 fold, and 99.62 % at the depth of 4X, 98.57 % at the depth of 10X, and 96.50 % at the depth of 20X of targeted bases were covered. For bioinformatic analysis, we focused on variants in coding regions. Variants in individuals and their parents with a MAF < 0.005 were filtered using ten databases: the 1000 Genomes Project, HapMap, EVS, Wellderly, and ExAC_v0.3 databases; and five in-house databases from BGI. After completing this filtering process, we identified twelve genes with variants shared by the two cases (Table 2). Among the twelve genes, the hearing loss-related gene *ACTG1* was identified. Considering the WES results, the prediction results using SIFT, PolyPhen, MutationTaster, MutationAssessor, LRT, FATHMM, GERP+, PhyloP, SiPhy, GERP, and phastCons (Table 3); the gene pathways; and their expression in the human fetal cochlear EST database, the variants in *COL6A1* were then tested using Sanger sequencing. The variant in *COL6A1* did not cosegregate in the affected and unaffected individuals. Therefore, *ACTG1* was identified as the gene associated with the family. The whole exome sequencing result concurred with that oftargeted sequencing of known deafness genes.

**Table 2:** Candidate gene variants found by NGS

	Gene	Variants	Protein change	case-III:2	case-II:1	control-II:2
1	*LRP2*	HT c.475 T > G	p.F159V	'0/1,35,27'^a^	'0/1,36,21'	'0/0,25,0'
2	*TTC14*	HT c.1298 T > C	p.L433S	'0/1,34,39'	'0/1,11,19'	'0/0,21,0'
3	*LYSMD3*	HT c.691A > T	p.I231L	'0/1,52,59'	'0/1,40,32'	'0/0,33,0'
4	*PROB1*	HT c.2552C > T	p.P851L	'0/1,9,3'	'0/1,2,6'	'0/0,10,0'
5	*DLD*	HT c.1189A > G	p.K397E	'0/1,36,31'	'0/1,30,31'	'0/0,23,0'
6	*RAD52*	HT c.767C > T	p.A256V	'0/1,21,23'	'0/1,19,20'	'0/0,21,0'
7	*UMOD*	HT c.691C > T	p.L231F	'0/1,22,21'	'0/1,40,26'	'0/0,27,0'
8	*ACTG1*	HT c.638A > G	p.K213R	'0/1,54,84'	'0/1,45,52'	'0/0,110,0'
9	*CDH7*	HT c.1427A > G	p.N476S	'0/1,35,39'	'0/1,14,22'	'0/0,21,0'
10	*ATCAY*	HT c.401 T > C	p.M134T	'0/1,40,39'	'0/1,40,39'	'0/0,21,0'
11	*XRN2*	HT c.1696 T > C	p.Y566H	'0/1,30,28'	'0/1,10,9'	'0/0,21,0'
12	*COL6A1*	HT c.457C > G	p.L153V	'0/1,175,155'	'0/1,171,154'	'0/0,20,0'

*HT* heterozygous; ^a^'0/1,35,27' 0 indicates the reference base, 1 indicates the first variant, 35 indicates the count of reads supporting the reference base, 27 indicates the count of reads supporting the variant base

**Table 3:** Pathogenicity Assessment in Silico of ACTG1 c.638A > G (p.K213R)

Tools	Pathogenicity	Functional Prediction Scores/Conservation scores
SIFT	Damaging	0
PolyPhen	Probably damaging	0.947
MutationTaster	Disease causing	0.999324
MutationAssessor	Deleterioius	4.105
LRT	Deleterioius	0
FATHMM	Deleterioius	−4.81
GERP+		4.56
PhyloP	Not conserved	1.72
SiPhy		12.999
GERP		4.56
phastCons	Conserved	1

### Structural modelling of p.K213R

**Fig. 3:**
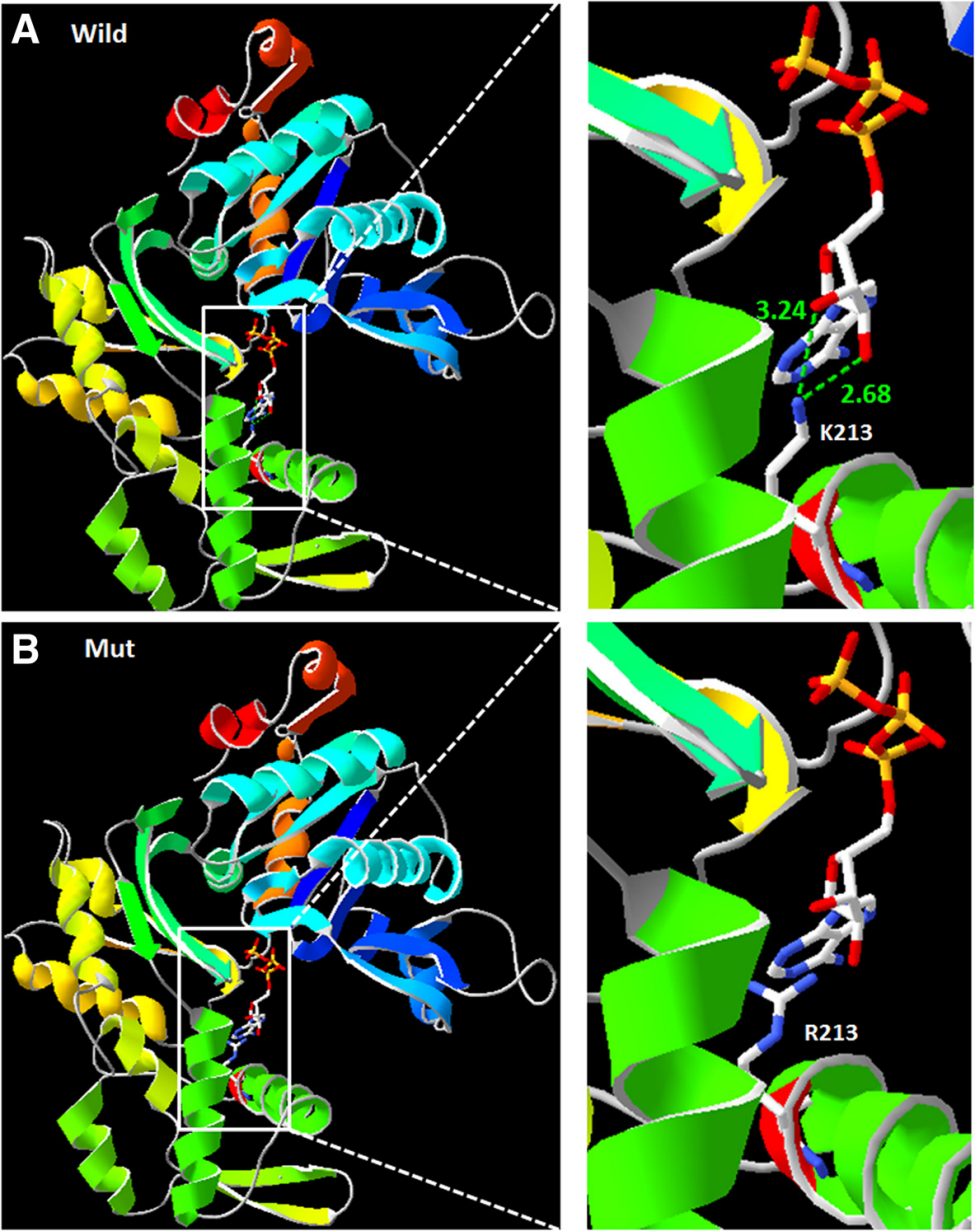
Structure of wild-type and mutant ACTG1. **a** K213 in the wild-type protein has two hydrogen bonds that interact with ATP. **b** Mutant R213 has lost its hydrogen bonds and does not interact with ATP

A molecular model of γ-actin was constructed based on the crystal structure of the heterodimer (PDB ID: 3ub5A). The constructed model covered the target sequence of *ACTG1* (residues 6–375). The sequence identity between the target and template was 99.73 %, higher than the average 25.00 %. Quality of the model were evaluated and fixed by Verify 3D and the results showed 99.46 % of the residues had an averaged 3D-1D score > =0.2 (pass). Using Swiss-PdbViewer 4.1, the mutation was predicted to lose two hydrogen bonds (2.68A, 3.24A) and influence the interaction with ATP due to the substitution of lysine by arginine (Fig. 3). MUpro software predicted that this mutation decreased the stability of the protein structure.

These data together with the clinical presentation of the affected individuals clearly indicate that the *ACTG1* c.638A > G (p.K213R) mutation was the genetic cause of ADNSHI in this family.

## Discussion

The *ACTG1* gene encodes γ-actin, which is a cytoskeletal protein abundantly expressed in the sensory hair cells of the cochlea [8, 9]. *ACTG1* is linked to the DFNA20/26 locus on chromosome 17q25.3. *ACTG1* is predicted to be essential for the shape and function of the stereocilia of the hair cells [10]. Recently, several exome sequencing studies were carried out and *ACTG1* mutations were identified as causes for either deafness or Baraitser-Winter syndrome. Park G et al. designed a multiphasic analysis of copy number variation, linkage, and single nucleotide variation of whole exome sequencing data for the efficient discovery of mutations causing nonsyndromic hearing loss, and selected a novel variant p.M305T in *ACTG1* as a disease-causing variant in a three generation pedigree [11]. Using whole-exome sequencing, Rivière JB et al. identified de novo missense mutations in the cytoplasmic actin-encoding genes *ACTB* and *ACTG1* in Baraitser-Winter syndrome (a disorder characterized by distinct craniofacial features, ocular colobomata and neuronal migration defect) patients [12]. Mutations in γ-actin cause hearing loss mainly by impairing the function and/or viability of hair cells [10, 13, 14]. To date, the following 12 missense mutations in *ACTG1* have been reported to cause ADNSHI: p.D51N, p.T89I(rs28999111), p.K118N(rs267606630), p.K118M(rs104894544), p.I122V(rs281875330), p.D187H, p.E241K(rs267606631), p.P264L(rs104894546), p.T278I(rs28999112), p.M305T, p.P332A(rs104894545), and p.V370A(rs104894547) [3, 10, 11, 13–17]. All are located in exons 3, 4, 5, and 6 of *ACTG1* (Table 4).

**Table 4:** Overview of *ACTG1* mutations described in DFNA20/26

No.	Mutation	Protein change	Exon	Sub-domain	Origin	Age of onset	Reference
1	c.151G > A	p.D51N	3	2	Dutch	1st decade	[15]
2	c.266C > T	p.T89I	3	1	American	3rd decade	[13]
3	c.353A > T	p.K118M	3	1	American	1st or 2nd decade	[13]
4	c.354G > C	p.K118N	3	1	Spanish	1st or 2nd decade	[16]
5	c.364A > G	p.I122V	4	1	Chinese	1st decade	[3]
6	c.559G > C	p.D187H	4	4	Korean	1st decade	[17]
7	c.638A > G	p.K213R	4	4	Chinese	2nd decade	Present study
8	c.721G > A	p.E241K	4	4	Spanish	1st decade	[16]
9	c.791C > T	p.P264L	4	4	American	1st or 2nd decade	[13]
10	c.833C > T	p.T278I	5	3	Dutch	1st or 2nd decade	[14]
11	c.914 T > C	p.M305T	5	3	Korean	3rd decade	[11]
12	c.994C > G	p.P332A	6	3	American	2nd decade	[13]
13	c.1109 T > C	p.V370A	6	C-terminal	Norwegian	1st or 2nd decade	[10]

γ-Actin comprises four subdomains (subdomains 1–4). The novel missense mutation p.K213R, which is caused by a c.638A > G transversion, results in the substitution of lysine for arginine in subdomain 4, which also causes the mutations p.D187H, p.E241K, and p.P264L. Even minor changes in this domain may lead to major effects on the structural stability of the actin polymer [18]. For p.D187H, it has been suggested that the mutation could collapse the polymerisation–depolymerisation balance of microtubules, leading to the destruction of cellular homeostasis in normal hair cells [19]. The p.E241K mutation was shown to lead to abnormal formation of thick randomly oriented action filament bundles [16]. Mice with the p.P264L mutation develop hearing loss concomitant with loss of the shortest row of actin stererocilia in hair cells [20]. These findings suggest that mutations in subdomain 4 affect normal hair cells.

Mutations in *ACTG1* have been associated with DFNA20 and DFNA26. Patients with DFNA20 and DFNA26 disorders display sensorineural hearing loss, initially only at high frequencies and steadily progressing to include all frequencies [21, 22]. Distortion product otoacoustic emission data show a cochlear lesion [21]. Phenotypic differences have been shown between previously reported DFNA20/26 families, in which tinnitus was not a feature, and the p.D51N mutation family, which is associated with tinnitus [15]. Tinnitus was not found in the present study.

The age of onset of hearing loss, which ranges from the first to third decade of life, differs among the different mutations in *ACTG1*. The age of onset of hearing loss caused by mutation p.D51N located in subdomain 2 of γ-actin is during the first decade of life [15]. And that associated with mutations p.T89I, p.K118M, p.K118N, and p.I122V located in subdomain 1 of γ-actin [3, 13, 16] as well as mutations p.T278I, p.M305T, and p.P332A located in subdomain 3 [11, 13, 14] ranges from the first to third decade. The average age of onset of hearing loss associated with mutations p.D187H, p.E241K, and p.P264L located in subdomain 4 is from the first to second decade of life [13, 16, 17] (Table 4).

In the present study, the age of onset of hearing loss for the affected family members excluding III:2 was during the second decade of life. III:2 was a 13-year-old boy who was born with hearing loss; his pure-tone average threshold for four frequencies (0.5, 1.0, 2.0, and 4.0 KHz) were 61.25 and 56.25 dB HL for the right and left, respectively. Interestingly, homozygous c.109G > A (p.V37I) in *GJB2* and heterozygous c.638A > G (p.K213R) in *ACTG1* were found in III:2, which differed from the other affected family members (Table 1). The c.109G > A (rs72474224) mutation is very common, although its pathogenicity remains controversial. However, it was previously reported that *GJB2* homozygous c.109G > A was considered to be a pathogenic mutation causing moderate to profound deafness [23]. In one study, c.109G > A was the second most frequent mutation among 126 south Chinese patients with NSHI carrying a homozygous or compound heterozygous *GJB2* pathogenic mutation (18.0 %, 126/701) [24]. In another study, the c.109G > A mutation had an allelic frequency of 6.7 % (185/2744) in Chinese Han patients, and this frequency was significantly higher than that in the control population (2.8 %, 17/602; P = 0.0003) [25]. These results support the notion that the *GJB2* recessive mutation c.109G > A should be considered a pathogenic mutation. Digenic mutations resulting in hearing loss have been reported in previous studies [5–7], but all exhibited recessive/recessive inheritance; a homozygous mutation associated with DFNB co-acting with a heterozygous mutation associated with DFNA is rare. In this study, III:2 carried homozygous c.109G > A in *GJB2* and heterozygous c.638A > G in *ACTG1*. These digenic mutations in III:2 showed an earlier age of onset than in the other affected family members. The audiogram of III:2 showed hearing loss at all frequencies, which was a more severe phenotype than that in III:4, although both of these family members were of the same age (Fig. 1e, g). We reviewed literatures on the phenotype of *GJB2* c.109G > A homozygous mutation and found that the onset age of hearing loss in those cases carrying the c.109G > A homozygous mutation varies from born to 13 years old. And the extent of hearing impairment is mainly mild to moderate except 4 cases. For the exception, one case showed progressive hearing loss reaching bilateral severe sensorineural hearing loss (85–90 dB) at 4 years old, another case progressed to severe hearing loss at the age of 2 years old, and the other two showed moderately severe sensorineural hearing loss (Table 5). These observations suggest that 1) additive effect of the *GJB2* and *ACTG1* gene defects might play a role in the phenotype in III:2; 2) *GJB2* might have synergized with *ACTG1* to result in a more severe phenotype in III:2.

**Table 5:** Phenotype of *GJB2* c.109G > A homozygous mutation

No.	Audiology findings	Diagnosed age	Reference
1	Mild to moderate hearing loss		
2	Mild sensorineural hearing loss	6 years	[33]
3	Mild to moderae hearing loss		[34]
4	Slight/mild sensorineural hearing loss		[35]
5	Bilateral high-frequency sensorineural hearing loss (40–60 dB)	4 years	[36]
6	Bilateral sensorineural hearing loss (85–90 dB),progressive: 55–60 dB at 2 years, 60–65 dB at 3 years,60–80 dB at 4 years	2 years	[36]
7	Bilateral sensorineural hearing loss (40–50 dB)	27 months	[36]
8	Mild to moderate high-frequency sensorineural hearing loss		[37]
9	Bilateral mild-moderate sensorineural hearing loss	8 years	[38]
10	Bilateral mild-moderate sensorineural hearing loss	3 years	[38]
11	Bilateral mild sensorineural hearing loss	13 years	[38]
12	Bilateral mild sensorineural hearing loss	3.5 years	[38]
13	Bilateral mild sensorineural hearing loss	born	[38]
14	Bilateral mild-moderate left ear and mild right ear		[38]
15	Bilateral mild	5 years	[38]
16	Bilateral mild high frequency	born	[38]
17	Moderate hearing impairment progressed to severe	2 years	[38]
18	Bilateral moderate hearing loss in high frequencies	4 years	[38]
19	Bilateral mild to moderate hearing loss	2 years	[38]
20	Bilateral moderate hearing loss	12 years	[38]
21	Bilateral mild hearing impairment		[38]
22	Bilateral moderately severe		[38]
23	Bilateral mild to moderately severe hearing loss		[38]

## Conclusions

In this study, by using targeted sequencing of known deafness genes, we identified a novel mutation, c.638A > G (p.K213R), in *ACTG1* in a Chinese autosomal dominant deafness family. We found one affected family member with a heterozygous c.638A > G mutation in *ACTG1* and a homozygous c.109G > A mutation in *GJB2*. To our knowledge, this is a rare DFNA family in whichone individual was affected by both a dominant mutation and a recessive mutation in two different genes. The more severe hearing phenotype was suggested to be resulted from these digenic mutations. This rare hereditary mode should be considered in clinical genetic diagnosis and counselling. Our results not only add to the theoretical basis of hereditary hearing loss, but will promote the translation of deafness gene capture and NGS in otology clinics.

## Methods

### Ethics statement

This study was approved by the Chinese PLA General Hospital Research Ethics Committee. Fully informed written consent for participation and publication of clinical data was attained from each subject or their guardians when the age of subjects <18 years old.

### Clinical data

A three-generation Chinese family with eight affected members and five unaffected members from Sichuan Province was evaluated. The medical history of each family member was obtained using a questionnaire (Additional file 1) that included the degree of hearing loss, age of onset of hearing loss, progression of hearing loss, symmetry of hearing loss, use of hearing aids, presence of tinnitus, pathological changes in the ear, infection, ototoxicity, noise exposure, and other relevant clinical manifestations to understand the otologic manifestation and exclude any history of other diseases and environmental factors. The proband underwent a number of clinical tests including general physical examinations, chest X-rays, brain MRI, and temporal bone CT. No abnormalities were detected in these tests, thus excluding the possibility that the hearing loss in this family was syndromic.

All genomic DNA (gDNA) was extracted from peripheral blood using a blood DNA extraction kit according to the protocol provided by the manufacturer (TianGen, Beijing, China).

### Audiometric analysis

Pure-tone audiometry with air and bone conduction was performed according to standard protocols in a sound-controlled room at frequencies ranging from 250 to 8000 Hz. Audiograms were available for six of the eight affected family members and for the one unaffected member.

### Deafness gene capture and Illumina library preparation

Among the affected family members, mutations in the common deafness genes *GJB2*, *SLC26A4*, and *mtDNA 12SrRNA* mutations were excluded, with the exception of homozygous c.109G > A in *GJB2*, which was found in III:2. Targeted NGS was then used to sequence 129 known deafness genes (http://www.otogenetics.com/forms/Deafness_v3_gene_list.pdf).DNA specimens from five patients and two normal hearing members of the family were sequenced by Otogenetics Corporation (Atlanta, GA, USA) using next-generation sequencingwith the Illumina platform. The quality of gDNA was examined by checking the optical density ratio (260/280 ratio) and performing gel electrophoresis imaging. High-molecular weight gDNA (approximately 3 μg) was fragmented ultrasonically using a Covaris E210 DNA shearing instrument (Covaris, Inc., Woburn, MA, USA) to an average size of 300 base pairs (bp). The Covaris protocol was a 3-min total duration, duty cycle of 10 %, intensity of 5, and 200 cycles per burst.

Exons and their flanking 50 bp from 129 known human deafness genes were selected for capture and NGS sequencing using an Illumina HiSeq2000. Hybridisation probes of 0.5 to 1.6 kilobase pairs (kb) were generated for these genes from either cDNA clones of the genes or by polymerase chain reaction (PCR) amplification from targeted gDNA regions. To ensure reliable capture of shorter exons, we specifically generated longer hybridisation probes from gDNA for those exons that were shorter than 50 bp by including approximately 100 bp genomic DNA flanking the exons on both sides. All PCR products (10 ng each) were purified using the QIAquick PCR Purification Kit (Qiagen, Valencia, CA, USA) before use. Further details of the capture probe validation and preparation can be found in a previous report [26].

Fragmented gDNA libraries for Illumina GAII sequencing were prepared using the NEBNextTM DNA Sample Prep Master Mix set (E6040; NEB Biolab, Ipswich, MA, USA). End repair of DNA fragments, addition of a 3′ adenine (A), adaptor ligation, and reaction clean-up were performed according to the manufacturer’s protocol. The libraries were purified and size-selected using the AMPure DNA Purification kit (Beckman Agencourt, Danvers, MA, USA). The ligated product (20 ng) was amplified over 14 PCR cycles using the Illumina PCR primers InPE1.0 and InPE2.0 and indexing primers according to the manufacturer’s instructions.

For targeted enrichment of deafness genes, the Illumina library DNA was purified using a QIAquickMinElute column and eluted into 50 μL hybridisation buffer (Roche NimbleGen, Madison, WI, USA). The barcoded Illumina gDNA libraries (5 μg) were incubated in 16 μL hybridisation buffer on the surface of hybridisation glass slides on a hybridisation station (BioMicro Systems, Inc., Salt Lake City, UT, USA) at 42 °C for 72 h. Nonspecific DNA fragments were removed after a series of six washing steps in washing buffer (Roche NimbleGen, Madison, WI, USA). The DNA bound to the probes was eluted by a 10-min incubation with NaOH (425 mL, 125 mM). The eluted solution was transferred to a 1.5-mL Eppendorf tube containing 500 μL neutralisation buffer (Qiagen’s PBI buffer). The neutralised DNA was desalted and concentrated on a QIAquickMinElute column and eluted into 30 μL EB buffer. To increase the yield, we typically amplified 5 μL eluted solution by 12 cycles of PCR using the Illumina PCR primers InpE1.0 and 2.0. Enrichment of the targeted deafness genes was examined by comparing the growth curves of captured and noncaptured samples during quantitative PCR [26]. Twelve barcoded libraries of captured samples were pooled, and paired-end Illumina sequencing was performed using the Illumina HiSeq system (Illumina, San Diego, CA, USA). Details of the bioinformatic analysis methods used have been published previously [26].

The sequence data were mapped with BWA (0.7.4) against the human reference genome index (hg19), and then analyzed with Picard to remove duplicates from the mapped reads. Variants in the data (SNPs/indels) were called with SAMtools (0.1.19) across the genome and exported in VCF format; 516.7 ± 28.5 variants were obtained per sample. All of the variants in the target regions were selected based on the bed file provided by Otogenetics, and then annotated with ANNOVAR and the internal mutation database to get information on the impact of each variant, predicted functional changes, 1000 Genome Project population allele frequency, and associated diseases, if applicable. Variants with known disease associations, a deleterious functional impact, or aMAF(Minor Allele Frequency) <0.04 were selected as candidate mutations for analysis and validation; 20.4 ± 5.4 variants were obtained per sample. To identify the pathogenic mutation, a cosegregation analysis of the family members and an in-house database of 481 Chinese normal hearing controls from Otogeneticswas applied.

### Whole exome sequencing

Exome capture was performed in the proband and his parents by BGI–Shenzhen using NimbleGen SeqCap EZ Human Exome Library v3.0 (Roche NimbleGen, Inc., Madison, WI, USA) according to the manufacturer’s protocols, and sequencing was performed using a HiSeq2000 platform (Illumina, San Diego, CA, USA). Illumina base calling Software 1.7 was used with default parameters to process the raw image files and to sequence the individual products as 90-bp paired-end reads. The sequenced reads were aligned to the human genome reference (UCSC hg19 version, build37.1) using SOAP aligner/SOAP2[27]. SNP or indels were called using Soapsnp [28] software and BWA [29], respectively. The alignment results were identified using GATK [30] to identify the breakpoints.

### Sanger sequencing

After filtering against multiple databases, Sanger sequencing was used to determine whether any of the potential mutations in known genes causing ADNSHI co-segregated with the phenotype in this family. Direct PCR products were sequenced using Bigdyeterminator v3.1 cycle sequencing kits (Applied Biosystems, Foster City, CA, USA) and analysed using an ABI 3700XL Genetic Analyzer.

### Mutational analysis

Segregation of the mutations was evaluated in all family members. Genotyping for c.638A > G was performed by PCR and detected by bidirectional sequencing of the amplified fragments using an automated DNA sequencer (ABI3100); the primers were 5′-CAGAGCCCTCCCTTAGTGAT-3′ and 5′-CGAGGCTACAGCTTCACCAC-3′. Nucleotide alterations were identified by sequence alignment with the *ACTG1* GenBank sequence (NG_011433) using Genetool software.

### Multiple sequence alignment

Multiple sequence alignment was performed across 15 species using ClustalW2 online (http://www.ebi.ac.uk/Tools/msa/clustalw2/).

### Model building and structure-based analysis

Three-dimensional modelling of the human wild-type and p.K213R mutation was performed using SWISS-MODEL [31], an automated homology modelling program (http://swissmodel.expasy.org/workspace/). We used the automatic modelling approach to model the complete human *ACTG1* protein, including its 375 amino acids (NP_001186883.1) with or without the mutations. Data obtained from the homology models were visualised using Swiss-PdbViewer 4.1. Quality of the structure model were assessed by Verify 3D.

#### Availability of supporting data

Sequence read data of the affected subject (II:3) has been deposited into Sequence Read Archive ([32]; accession number SRP064631).

## Additional file

Additional file 1:
**Questionnaire for deafness.** (DOCX 14 kb)
