# Supplementary material for: Phenotypic Heterogeneity in a DFNA20/26 family segregating a novel ACTG1 mutation
Source: BMC Genet. 2016 Feb 1;17:33. doi: 10.1186/s12863-016-0333-1 (PMC4736096; doi:10.1186/s12863-016-0333-1)
Supplement: Additional file 1: — Questionnaire for deafness. (DOCX 14 kb) [file 12863_2016_333_MOESM1_ESM.docx]

**Questionnaire for deafness**

**1.General information**

Name:

Sex:

Age:

Email:

Phone number:

Address:

**2.General physical examinations**

**3.Family history of hearing loss**

Yes No

If yes, draw the pedigree tree.

**4.Usage of aminoglycosides**

Yes No

If Yes, state when, what dosage and by which way it was used (oral or injection.etc)

**5.Noise exposure history**

Yes No

If Yes, state **the possible noise intensity and the duration of exposure.**

**6.Hearing information**

(1) Age of onset of hearing loss:

(2) Degree of hearing loss: PTA R: PTA L:

(3) Progression of hearing loss:

Yes No

If Yes, state when it began and how it progressed.

(4) Use of hearing aids

Yes No

(5) Presence of tinnitus

Yes No

**7.Imaging examinations**

Temporal bone CT.

Inner ear water imaging

Brain MRI

**8. Other relative clinical manifestations**
